# Effectiveness of an organized colorectal cancer screening program on increasing adherence in asymptomatic average-risk Canadians

**DOI:** 10.1186/1472-6963-13-449

**Published:** 2013-10-29

**Authors:** Thomas J Charters, Erin C Strumpf, Maida J Sewitch

**Affiliations:** 1 Department of Epidemiology, Biostatistics and Occupational Health, McGill University, Purvis Hall 41, 1020 Pine Ave. West, Montreal, QC H3A 1A2, Canada; 2 Department of Epidemiology, Biostatistics and Occupational Health, McGill University, Leacock 418, 855 Sherbrooke St. West, Montreal, QC H3A 2T7, Canada; 3 Department of Economics, McGill University, Leacock 418, 855 Sherbrooke St. West, Montreal, QC H3A 2T7, Canada; 4 Division of Clinical Epidemiology, Research Institute of the McGill University Health Centre, 687 Pine Ave. West V-Building, Montreal, QC H3A 1A1, Canada; 5 Department of Medicine, McGill University, 687 Pine Ave. West V-Building, Montreal, QC H3A 1A1, Canada

**Keywords:** Colorectal neoplasms/diagnosis, Fecal occult blood test, Endoscopy, Mass screening, Program evaluation, Ontario, Canada

## Abstract

**Background:**

Colorectal cancer (CRC) is the third most commonly diagnosed cancer and second highest cause of cancer-related mortality in Canada. Despite the availability of screening services and establishment of guidelines, utilization of colorectal cancer screening in Canada remains low. In 2008, the province of Ontario launched *ColonCancerCheck,* an organized colorectal cancer screening program aimed at increasing CRC screening adherence. In this study, we adopt a quasi-experimental approach to estimate and describe the impact of *ColonCancerCheck* on screening behavior in the asymptomatic average risk population.

**Methods:**

Annual screening rates from the target population were estimated using five cycles of the Canadian Community Health Survey, a cross-sectional nationally representative survey of health status, healthcare use, and determinants of health in the Canadian population. We used a difference-in-differences design to measure the overall impact of *ColonCancerCheck* on past-year fecal occult blood testing (FOBT) and endoscopy in Ontario relative to the rest of Canada. Several verification tests validated the suitability of our model specification.

**Results:**

The difference-in-differences analysis shows that *ColonCancerCheck* increased FOBT screening in the average risk population by 5.2 percentage points (95% CI [3.2, 7.2]), an increase of 33% relative to pre-program screening rates. The program had no observed effect on endoscopy screening and we found no evidence that *ColonCancerCheck* differentially altered the screening practices of population sub-groups.

**Conclusions:**

Our findings suggest *ColonCancerCheck* has been successful at increasing use of FOBT in the asymptomatic average risk population.

## Background

Colorectal cancer (CRC) constitutes the second highest cause of cancer mortality and third most commonly diagnosed cancer in Canada [1]. In 2013, the incidence of CRC in Canada is projected to be 60 per 100,000 in males and 40 per 100,000 in females, with approximately 4200 women and 5000 men dying from it [1]. The demographic shift towards an older population has increased CRC incidence and mortality over the last thirty years [2]. Despite opportunities for early detection [2], CRC tends to be diagnosed at advanced cancer stages [3], which are associated with both lower probabilities of survival [4] and greater costs to the healthcare system [5].

CRC screening guidelines have been developed and adapted based on the recognition that early detection and treatment can significantly reduce CRC morbidity and mortality [6]. These guidelines were based on existing knowledge of the best means for early detection of cancer [7-9] (since updated [10]) with screening recommendations typically calling for annual or biennial screening by a noninvasive stool-based test such as a fecal occult blood test (FOBT) with follow-up with endoscopic tests such as flexible sigmoidoscopy or colonoscopy. A Cochrane systematic review of several large randomized trials approximating population-based screening strategies incorporating biennial fecal occult blood tests estimated that screening reduces CRC mortality by 15% [11]. Population-based screening is generally thought to be cost effective [12,13] and is supported by Canadian [14,15] and other [16] microsimulation models.

Screening rates for CRC in Canada remain quite low despite the presence of screening guidelines, evidence of screening effectiveness, and universal health insurance coverage for these procedures [17]. In 2003, adherence to screening guidelines for FOBTs among average risk, target-age individuals was only 15% [18], increasing to 23% in 2008 [19]. Screening rates have also been observed to vary by province, with Ontario historically having higher rates than much of the rest of Canada [19]. By contrast, 73% of Canadian women aged 50–69 reported mammogram adherence in this time period [20]. Evidence suggests that both Canadian patients [21] and physicians [21,22] were unknowledgeable of, or non-adherent to, recommended screening protocols for CRC. Outside of Ontario and in Ontario prior to 2008, CRC screening broadly followed the procedures as outlined in the national guidelines [7-9], albeit in an opportunistic or individualistic manner which gave rise to concerns of underutilized or inappropriate screening and lack of quality assurance [23].

In order to address low adherence to screening guidelines, Ontario’s Ministry of Health and Long Term Care launched an organized population-based colorectal cancer screening program called *ColonCancerCheck* in March of 2008 [23]. *ColonCancerCheck* targeted adults ages 50 and older with the goals of reducing CRC mortality and enhancing the involvement of physicians in patient screening. The program utilized existing screening recommendations for biennial FOB testing with endoscopy follow-up for average risk adults with colonoscopy being recommended for those of high risk. It also introduced outreach and organizational frameworks designed to increase screening adherence and educate both the public and health care providers about proper screening protocols.

To increase adherence to screening practices in the target population, *ColonCancerCheck* directed educational media campaigns to physicians through information kits and counseling manuals, and to the public, through television, websites, posters, pamphlets, and street teams at public events. With primary care providers (PCPs) taking the central role of distributing kits and following up with patients, arrangements were made for those without PCPs to obtain kits through pharmacies or through calling a 1–800 number. Contracts were established with laboratories to process tests and develop quality-control standards. A single brand of FOBT kit with sufficient accuracy and modest dietary restrictions was selected and used by all participating laboratories to help ensure quality, consistency, and interpretability of results. FOBT kits were handed out with instructions and post-paid envelopes to be completed at home and mailed to central laboratories for processing. An organizational framework was developed for screening invitations and results letters with pilot programs for invitation-based outreach in development. PCPs would be informed of their patient’s test results and follow-through by arranging colonoscopies if positive, with negative results leading to notification and recall for testing in two years. Incomplete and unreadable tests would be corrected with instructions returned for re-tests [23]. Prior to the launch of the program, FOBT kits were sent out to over 10,000 physician and nurse practitioner offices and 3000 pharmacies [23].

Some evidence suggests increased screening in Ontario after the program’s introduction. Health insurance claims indicate that 30% of Ontarians aged 50–74 had at least one complete FOBT in the previous two years in 2007–8 (inclusive) compared to 20% in 2005–6 [23]. Survey data corroborates this, suggesting FOBT adherence rose from 21% to 31% from 2005 to 2008, while endoscopy test adherence rose from 24% in 2005 to 30% in 2008, in those 50 and older [19]. However, these findings are insufficient to draw conclusions concerning the impact of *ColonCancerCheck* since there was a general trend of increased screening across Canada with increases observed in several provinces without screening programs during this time period [19]. Similarly designed programs were under various stages of development in Manitoba [24] and Alberta [25] during this time, with Manitoba instigating a pilot program for CRC screening in two health regions. However, no province-wide CRC screening programs outside of Ontario were implemented during the study period.

The purpose of the present study was to measure and describe the impact of *ColonCancerCheck* on CRC screening in the asymptomatic, average risk population in Ontario. We used a quasi-experimental design and a difference-in-differences model, which measures the change in screening rates in Ontario relative to the change in the rest of Canada. This model has been used extensively to measure the impacts of health care policies, including the effects of public reporting of hospital performance on mortality [26] and the effects of an intensified diabetes management program [27]. A similar model was also used to measure the effects of mandated insurance coverage for endoscopy screening in the U.S. on CRC screening rates among adults aged 50–64 [28].

## Methods

### Data

This study used the confidential microdata files from the Canadian Community Health Survey (CCHS) in 2003, 2005, 2007, 2008, and 2009 available from the Statistics Canada Research Data Centres. The CCHS is a cross-sectional, nationally representative survey of individuals aged 12 and older living in private dwellings in Canadian provinces and territories. Persons living on Indian reserves, on Crown lands, in institutions, in remote regions, or serving in the Canadian Forces, are excluded from the sample frame [29]. The CCHS was initially a biennial survey although was revised in 2007 to become an annual survey with smaller samples. During the study period, the sample size ranged from 61,679 in 2009 to 135,573 in 2003 with response rates ranging from 73.2% in 2009 to 80.7% in 2003.

The primary outcome in this study was CRC screening in the past year, which was assessed based on questions pertaining to FOBT and endoscopy tests and time since their receipt. Respondents were coded as having been screened for CRC in the past year if they reported receiving FOBT and/or endoscopy “less than one year ago”. FOBT and endoscopy were described by the CCHS to interviewees as follows: “An FOBT is a test to check for blood in your stool, where you have a bowel movement and use a stick to smear a small sample on a special card” and endoscopy: “A colonoscopy or sigmoidoscopy is when a tube is inserted into the rectum to view the bowel for early signs of cancer and other health problems” [30]. During the study period, the Canadian screening guidelines were consistent with respect to FOBT and endoscopy use. Past-year receipt of screening was used in order to minimize measurement error and exposure misclassification. The independent variable was exposure to *ColonCancerCheck*. Exposed individuals were CCHS respondents residing in Ontario in 2008 and 2009, while unexposed individuals lived in Ontario in 2003, 2005, and 2007 or in another province in any survey year. Details on socio-demographic characteristics, health status, and the medical services use of respondents were included as potential confounders.

This study is a secondary analysis of data collected by Statistics Canada. The CCHS, which operates under the provisions of the *Statistics Act. 1970-71-72. C.15, s.1*[31], safeguards release of information which could disclose the identity of any person or organization and obtains informed consent from interviewees with permission granted from parents/guardians for youth interviewees [29]. As a secondary analysis of anonymous data, our study falls under Article 2.4 of the Canadian Tri Council Policy Statement on Ethical Conduct for Research Involving Humans: “REB review is not required for research that relies exclusively on secondary use of anonymous information, or anonymous human biological materials, so long as the process of data linkage or recording or dissemination of results does not generate identifiable information” [32]. Because the database does not contain identifiable information, the McGill University Faculty of Medicine Institutional Review Board did not require ethics review and approval. This study was approved by the Social Sciences and Humanities Research Council of Canada on March 23, 2011 (Project 11-SSH-MCG-2659).

### Population

We sought to approximate the principal target population of *ColonCancerCheck*[23] of asymptomatic, average-risk adults aged 50–74. We removed identifiable high risk respondents from the sample including those who reported screening with either FOBT or endoscopy due to family history of CRC, or as a follow-up to treatment of CRC. Those reporting bowel disease such as colitis or Crohn’s disease were also excluded. We excluded respondents living in Canadian territories from the control group, as the geographically remote territories have limited comparability to Ontario and were noted by others [19] to yield unreliable screening estimates. While Ontario and Newfoundland and Labrador were included in every survey year, the control group consisted of an unbalanced panel of provinces since CRC screening was an optional module in which most provinces participated only in selected survey years (Table 1).

**Table 1:** Participation in module on CRC screening questions by year

**Province**	**2003**	**2005**	**2007**	**2008**	**2009**
**Newfoundland and Labrador**	**●→**	**●→**	**●→**	**●→**	**●→**
**Prince Edward Island**		**●→**	**●→**	**●→**	**●→**
**Nova Scotia**		**●→**		**●→**	**●→**
**New Brunswick**		**●→**		**●→**	**●→**
**Quebec**				**●→**	
**Ontario**	**○→**	**●→**	**●→**	**●→**	**●→**
**Manitoba**				**●→**	
**Saskatchewan**	**○→**		**●→**	**●→**	**●→**
**Alberta**				**●→**	
**British Columbia**	**●→**			**●→**	

An open circle indicates not all health regions were surveyed for this province; a closed circle indicates all health regions were surveyed. We found no evidence of disproportional representation of health regions which would influence the screening outcome measures, so the subsample of health regions used in the 2003 cycle was thought to be a suitable representative of the province.

### Analysis

We combined five cycles of the CCHS to assess temporal trends in screening by province. Given consistency in the form and type of questions, sampling frames, and population of interest over time, pooling across years was felt to be appropriate. To assess whether the other provinces were an appropriate control group for Ontario, we analyzed the difference in means of selected covariates in Ontario and other Canadian provinces in the pre-policy period. To estimate the effect of *ColonCancerCheck* on screening activities we employed the difference-in-differences (DD) model. The DD’s quasi-experimental framework takes the form of a fixed effects model suited to measuring the impact of a policy which varies at a group (provincial) level over time in a non-randomized setting [33]. The DD design is attractive as it removes bias due to temporal trends in screening, from confounders common to both the intervention and control groups and due to time-invariant differences between Ontario and other Canadian provinces.

We ran the following logistic regression:

$$\begin{array}{l} Y_{\mathrm{igt}}=\beta 0+\beta 1*\mathrm{Ontari}o_{g}+\beta 2*\mathrm{Post}\;\mathrm{Interventio}n_{t}\\ \;+\beta 3*\mathrm{Ontario}*\mathrm{Pos}t_{\mathrm{gt}}+\beta 4*\mathrm{cova}r_{\mathrm{igt}}+\epsilon_{\mathrm{igt}} \end{array}$$

Where *Y* is receipt of screening in the past year for individual *i* in province group *g* and time *t*. *β3* is the effect measure, representing the change in screening rates due to *ColonCancerCheck (CCC)* relative to the change in screening in provinces without the organized screening program. Indicator variables for the treatment group (Ontario) and for the post-intervention period (2008–09) serve as controls and adjust for fixed differences between the intervention and control provinces, and common trends over time, respectively. Covariates included indicators for each survey year and province, sex, age category (50–64, 65–74), geography (rural, urban location), self-rated health (poor, fair, good, very good, excellent), having a regular physician, ever having had a flu shot, a physical activity index score aggregating a number of leisure activities (active, moderately active, inactive), smoking status (regular, occasional, never), ethnicity (Caucasian, other), education (<secondary school, secondary school, some post-secondary, post-secondary), income (household quintiles standardized to national level), and the number of reported visits to a general practitioner in the past year (0–3, 4–10, 11–19, 20 or more). Further details on question methodology and operationalization may be found elsewhere [30,34].

Because the DD design examines relative changes over time, it requires that *trends in* screening, rather than *levels of* screening, be similar in the pre-intervention period. Therefore, under the assumption of parallel pre-intervention screening trends, two provinces with differing levels of screening prevalence are comparable. Having chosen an appropriate control group with similar pre-intervention trends and baseline characteristics, we interpret the intervention as being responsible for any deviation from these previously established trends [33]. To our knowledge, there were no program or budgetary actions initiated at the provincial level during the study time period which would have substantially impacted CRC screening rates other than *ColonCancerCheck,* and we therefore feel this assumption is valid.

We conducted a complete case analysis. Secondary analysis suggested that item non-response was not informative of the outcomes. All results were translated from logistic regression coefficients into predicted probabilities, and marginal effects were calculated on the risk difference scale with 95% confidence intervals. Using an approach recommended for the CCHS [35], we rescaled the original sample weights to account for differences in sample size across years by a factor of *nj/(n1 + n2 + …nj)* where *n* represents the sample size of each survey *1,2…j*[36]. The CCHS has a complex design including stratification, multiple stages of selection, and unequal selection probabilities [29]; therefore, bootstrap repeated replications [37] with pooling-adjusted weights were used to calculate variance estimates. All statistical analyses were performed using STATA version 11 [38].

### Model verification

We also performed three tests to verify the suitability of the DD model. The first was an extension of the DD model known as a difference-in-difference-in-differences model (DDD) which tests for the existence of factors that differentially affect screening rates in Ontario in the post-intervention period which could mistakenly be attributed as impacts of the *ColonCancerCheck* program. This analysis adds a further interaction to the *β3* term with several variables separately, noted previously [19] to be important modifiers of screening practices. These included having a regular doctor, being aged 65–74 years old, being “inactive”, reporting ever having had a flu shot, and being aged 35–49, this last category being less likely to screen. The DDD design has previously been used connecting variations in states’ introduction of the Medicaid program and labor force participation of eligible women [39]. Second, we conducted a DD falsification analysis using pre-CCC survey years with 2007 as the simulated post-intervention period. Under this test, a significant result would indicate the presence of residual confounding attributable to nonparallel trends between groups. Third, we used an alternative dependent variable, “ever having had a flu shot”, to check for bias plausibly linked to unmeasured changes between study groups through expansions to new users of flu vaccines. Since *ColonCancerCheck* should have no impact on use of flu shots, a significant result would suggest that the DD estimate is driven by larger systematic changes between health care systems within Canada.

## Results

The total sample based on province-years that administered the CRC screening module was 81,262, which was reduced to 58,142 after excluding individuals reporting screening due to family history, CRC treatment, or having colitis (10,030) and restricting the data for complete case analysis (13,090). Ontario contributed 19,888 and 11,293 observations and the control provinces 12,324 and 14,637 observations in the pre- and post-intervention periods, respectively.

**Figure 1:**
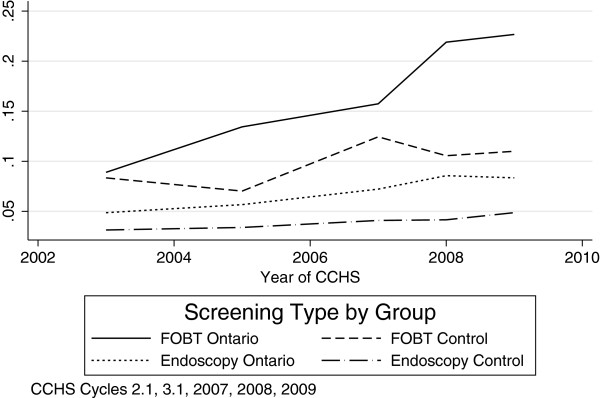
Past Year CRC Screening in Canada.

Comparisons between the intervention and control groups in the pre-intervention period generally show similarities across a range of measures, indicating the appropriateness of the control group. Notable differences include urban geography, ethnic diversity, flu shot uptake, and screening adherence (Table 2). Figure 1 indicates FOBT screening trends in Ontario rose more consistently than those in the control group, and jumped from 15.7% to 21.9% from 2007 to 2008. FOB testing trends in the control group are fairly similar to those in Ontario overall, despite an uncharacteristic decrease in 2005. FOBT use in the control group decreases from 12.4% in 2007 to 10.6% in 2008. Rates of past year endoscopy screening rose gradually in both groups, with little indication of a change in trajectory in 2008.

**Table 2:** Comparisons of study population in Ontario and other Canadian provinces (Control) 2003-2007

	**Control**	**Ontario**	**p**		**Control**	**Ontario**	**p**		**Control**	**Ontario**	**p**
				**Ethnicity**				**Leisure physical activity**			
**Socio-demographic**				Caucasian	91.5	85.5	0.000	Active	23.5	22.8	0.322
				Other	8.5	14.5		Moderately Active	26.8	26.8	0.995
**Age**								Inactive	49.6	50.4	0.400
50-64	74.0	73.2		**Country of birth**							
65-74	26.0	26.8	0.159	Canada	81.6	65.4	0.000	**Heavy drinking**^**b**^			
								Never	74.4	74.7	0.657
**Sex**				**Time in Canada since immigration**				<Once a Month	14.1	13.9	0.654
Male	53.0	52.0	0.136	Non-Immigrant	68.1	61.9	0.000	Once a Month	4.0	3.8	0.601
Female	47.0	48.0		20 or more	26.0	30.7	0.000	2-3 Times a Month	2.6	2.8	0.440
				10 to 19	3.2	4.8	0.001	Once a Week	3.1	2.6	0.058
**Marital status**				0 to 9	2.7	2.6	0.899	> Once a Week	1.8	2.2	0.058
Married	74.3	73.0	0.067								
Common-Law	4.1	4.7	0.082	**Health status**				**Health care use**			
Widowed	6.6	6.6	0.848								
Separated	2.6	2.7	0.560	**Self-perceived Health**				**Has regular MD**			
Divorced	7.3	7.6	0.395	Poor	3.6	3.7	0.730	Yes	93.3	94.4	0.007
Single, Never Married	5.2	5.4	0.614	Fair	11.7	11.4	0.593				
				Good	30.2	29.8	0.592	**Screening in past year: FOBT**			
**Highest level of education**				Very Good	36.3	35.9	0.581	Yes	8.4	13.5	0.000
< Secondary School	24.9	20.0	0.000	Excellent	18.2	19.3	0.109				
Secondary School	17.3	18.2	0.181					**Screening in past year: endoscopy**			
Some Post-Secondary	6.2	6.2	0.966					Yes	3.3	6.0	0.000
Post-Secondary	51.7	55.6	0.000	**Body mass index**							
				Underweight	1.0	1.1	0.442	**Ever had a flu shot**			
**Household income**^**a**^				Overweight	41.0	41.1	0.831	Yes	52.7	70.7	0.000
Quintile 1 (Lowest)	19.9	16.0	0.000	Obese-Class I	15.7	15.5	0.709				
Quintile 2	20.0	18.4	0.029	Obese-Class II	3.7	3.5	0.706	**No. GP consultations past year**			
Quintile 3	19.6	19.7	0.902	Obese-Class III	1.5	1.5	0.919	0 to 3	62.3	65.7	0.000
Quintile 4	18.9	20.1	0.080					4 to 10	30.4	28.9	0.062
Quintile 5 (highest)	21.7	25.8	0.000	**Health behaviours**				11 to 19	5.8	4.4	0.000
								20 or more	1.6	1.0	0.005
**Geography**				**Type of smoker**							
Urban	69.1	82.8	0.000	Daily	15.5	15.6	0.883	**Year**			
Rural	30.9	17.2		Occasional	2.7	3.0	0.397	2003	60.2	15.3	0.000
				Never	81.8	81.4	0.603	2005	30.2	53.6	0.000
								2007	9.6	31.1	0.000

N=30,484; Weighted N=1,468,619; All proportions weighted. ^a^Based on national composition excluding territories. ^b^Imbibing five or more drinks on one occasion.

Table 3 shows the results of the difference-in-differences regressions. We estimate a 5.2 [3.2, 7.1] absolute percentage point increase in the proportion of asymptomatic average risk individuals screened with FOBT in the past year due to *ColonCancerCheck*. This effect represents a significant marginal increase over significant temporal trends in FOBT screening and significant differences in screening between provinces, as indicated by the time and group coefficients, respectively. There were no effects found using this model pertaining to endoscopy outcomes 0.9 [−0.5, 2.3].

**Table 3:** Difference-in-differences estimates for fecal occult blood test and endoscopy outcomes

**Outcome**		**DD**^**a**^				**DD**^**b**^			
		**Marginal effect**	**95% CI**		**p**	**Marginal effect**	**95% CI**		**p**
**FOBT**	**Post intervention**	0.073	0.052	0.095	0.000	0.074	0.053	0.095	0.000
	**Ontario**	0.082	0.059	0.104	0.000	0.064	0.041	0.086	0.000
	Ontario*Post	0.050	0.030	0.070	0.000	0.052	0.032	0.071	0.000
**Endoscopy**	**Post intervention**	0.022	0.008	0.037	0.003	0.023	0.009	0.038	0.002
	**Ontario**	0.018	0.005	0.031	0.006	0.009	−0.004	0.022	0.182
	Ontario*Post	0.007	−0.007	0.021	0.301	0.009	−0.005	0.023	0.207

N = 58,142; Weighted N = 2,882,630. All results are weighted.

Dependent variable is self-report of having a FOBT or endoscopy (flexible sigmoidoscopy or colonoscopy) in past year.

^a^: controlled for year and province indicators.

^b^: controlled for year, province, sex, age category, geography, self-rated health, having MD, reporting flu shot, physical activity index, smoking status, ethnicity, education, income, #GP consultations past year.

The difference-in-difference-in-differences analysis allows us to test for effect measure modification to describe the impact of *ColonCancerCheck* and investigate whether it may alter the screening practices of population sub-groups. No evidence was found suggesting this is the case, or that the results are biased by any unmeasured underlying sample characteristics (Table 4). A near-significant change in association was related to endoscopic procedures and having a regular medical doctor in post-intervention Ontario, indicating a 6.0 [−0.7, 13.7] point marginal effect. However, this change did not coincide with an overall increase in endoscopy services and may relate to changes in mode of services delivery. Tables 5 and 6 indicate that the placebo tests did not find evidence of bias as evidenced by a lack of significant results for the Ontario*Post interaction term.

**Table 4:** Difference in difference in differences estimates for fecal occult blood test and endoscopy outcomes

**Outcome**		**DDD**			
		**Marginal effect**	**95% CI**		**p**
**FOBT**	ON*Post*MD	0.008	−0.095	0.111	0.883
	ON*Post*Ages65-74	0.028	−0.008	0.065	0.127
	ON*Post*Ages35-49^**a**^	−0.020	−0.052	0.012	0.214
	ON*Post*Inactive	0.007	−0.029	0.042	0.720
	ON*Post*Flu-Shot	0.010	−0.031	0.052	0.623
**Endoscopy**	ON*Post*MD	0.065	−0.007	0.137	0.077
	ON*Post*Ages65-74	0.002	−0.022	0.027	0.853
	ON*Post*Inactive	−0.003	−0.027	0.022	0.835
	ON*Post*Flu-Shot	−0.007	−0.035	0.021	0.628

N = 58,142; Weighted N = 2,882,630. Except ^a^: N = 98,023; Weighted N = 5,844,306. All results are weighted.

Dependent variables are self-report of having a FOBT or endoscopy (flexible sigmoidoscopy or colonoscopy) in past year. Group*Time terms are interacted with having a regular medical doctor, being aged 65–74 years, being aged 35–49 years, self-report of “inactive” on the Leisure Physical Activity Index, and reporting ever having had a flu shot. Control variables include year, province, Group, Time, Group*Time, sex, age category, geography, self-rated health, having MD, reporting flu shot, physical activity index, smoking status, ethnicity, education, income, #GP consultations past year, and Group* and Time* interactions with all of the characteristics listed above.

**Table 5:** Placebo test for incorrect policy implementation period

**Outcome**		**DD**			
		**Marginal effect**	**95% CI**		**p**
FOBT	Post intervention^a^	0.0875	0.0551	0.1200	0.000
	Ontario	0.0688	0.0439	0.0936	0.000
	Ontario*Post^a^	−0.0204	−0.0557	0.0148	0.256
Endoscopy	Post intervention^a^	0.0001	−0.0205	0.0208	0.989
	Ontario	0.0100	−0.0052	0.0253	0.198
	Ontario*Post^a^	0.0218	−0.0010	0.0447	0.061

N = 30,484; Weighted N = 1,468,619. All results are weighted. Dependent variable is self-report of having a FOBT or endoscopy (flexible sigmoidoscopy or colonoscopy) in the past year. Controlled for year, province, sex, age category, geography, self-rated health, having MD, reporting flu shot, physical activity index, smoking status, ethnicity, education, income, #GP consultations past year.

^a^: The post intervention period has been altered to equal 2007. Data from 2008–9 are not included.

**Table 6:** Placebo test for alternate dependent variable (Flu Shot)

**Outcome**		**DD**			
		**Marginal effect**	**95% CI**		**p**
Flu shot	Post intervention	0.0743	0.0453	0.1032	0.000
	Ontario	0.2588	0.2366	0.2810	0.000
	Ontario*Post	−0.0153	−0.0436	0.0131	0.291

N = 58,142; Weighted N = 2,882,630. All results are weighted. Dependent variable is self-report of ever having a flu shot. Controlled for year, province, sex, age category, geography, self-rated health, having MD, physical activity index, smoking status, ethnicity, education, income, #GP consultations past year.

## Discussion

We used a quasi-experimental causal approach to measure the effect of *ColonCancerCheck* on the past-year screening behavior of asymptomatic average risk adults ages 50–74. We found evidence that *ColonCancerCheck* increased screening adherence within the time frame studied. Past-year screening rates using FOBT increased by an absolute 5.16 [3.19, 7.12] percentage points, equivalent to a 33% increase relative to screening rates in Ontario in 2007. Our findings were robust to different model specifications. Additionally, we failed to find any evidence that past-year endoscopy screening rates were affected by the program. Subsequent tests of model suitability did not indicate evidence of effect modification by any of the factors investigated, or sources of bias. Differences in demographic and medical characteristics between the intervention and control groups were minor and accounted for by the DD model which controls for time-invariant differences across groups. Bias may result if underlying variables were to change alongside the intervention, although inter-group analyses and DDD models did not find any evidence of such interactions.

Although there has been some evidence for increases in FOBT use in Ontario in 2008 [19,23], this study is the first to estimate the proportion of these increases which can plausibly be attributed to *ColonCancerCheck*. To our knowledge, this is also the first study to analyze the direct impact of a CRC screening program using a quasi-experimental model. It is unsurprising that a program effect was only observed for FOB testing as the FOBT was the front line test for CRC screening [23] and is recommended to be administered more frequently than other screening tests. On the other hand, screening rates for colorectal cancer are much lower than for other cancers, so a priori it was not obvious whether the program would succeed in overcoming evident barriers to screening and increase screening rates.

In contrast, recommendations for endoscopy in our target group call for its use in screening at up to 10 year intervals [9] and as a follow-up test to positive FOBT results. Length of time to follow-up with endoscopy and low adherence for the procedure may account for our lack of observed effect. Results from the Ontario Pilot recorded median follow-up times from positive FOBT to endoscopy of 121 days in men and 202 in women [40]. Preliminary program results measured follow-up endoscopy within six months following a positive FOBT at 62.1% [23] with only 55% being performed within the target eight weeks [41]. Supply constraints and significant waiting times in the Canadian context [42] are other factors which could lead to delays. The time frame of this study was therefore likely insufficient to capture a potential increase in endoscopy use resulting from increased FOBT screening. Additionally, low follow-up may be explained by the fact that one third of FOB tests performed in Ontario in 2009–10 were outside of the *ColonCancerCheck* program and did not benefit from the program’s registry and follow-up processes [43]. The restrictions of the study period and the inability to assess program-specific clinical outcomes are inherent constraints of the study, and offer important avenues for future research.

We did not observe evidence of effect modification by having a regular physician for FOBT screening in post-intervention Ontario, which was somewhat surprising given the central role of primary care providers in the program [23]. One possible explanation is that outreach to individuals without PCPs was improved through the program. Secondary analyses [34] found similar increases in screening amongst those with a regular doctor in both Ontario and the other provinces over time, which supports the idea of temporal, rather than program-derived, influence of PCPs on patient screening.

There are several study limitations that should be addressed. First, because the colorectal cancer screening module was not asked in all provinces in all survey waves, we were required to use an unbalanced panel for our control group. This may have disproportionately represented Eastern Canada and biased screening trends in the pre-intervention period downward. This is a concern because, even though the DD and DDD designs can control for time-invariant group differences, alteration of the control group can distort trends and lead to differential results. In particular, the year 2005 indicated a decrease in proportion screened from the previous survey year from 8.34% to 7.03% for the FOBT outcome which was uncharacteristic of the overall trend. However, Table 5 indicates that the DD model is robust to unmeasured trends given the lack of an estimated effect in the pseudo-treatment period.

A second concern is residual confounding based on time-varying unmeasured factors in this observational study. To address this concern, we examined several interactions including having a regular doctor, being aged 65–74 years and 35–49 years, being “inactive”, and reporting ever having had a flu shot. These were chosen given their face validity and common citation in the literature as modifiers of screening. Given no evidence of effect modification by these factors, and no sources of bias found in any other verification test, it seems unlikely that other confounders would bias our estimates appreciably. Although past year outcomes were used to reduce exposure misclassification, the retrospective nature of the reporting likely results in some degree of misclassification and potential attenuation of the treatment effect. Underestimation of standard errors through auto-correlated residuals is common to DD [44], however the relatively short time series and independent nature of the dependent variables mitigates this. Measurement error in self-reported screening rates is another concern, with FOBT recall accuracy estimated to have a sensitivity of 82% and specificity of 78% [45]. Assuming that potential reporting errors are similar in the treatment and control groups given consistency in mode of collection and phrasing of questions [35], we expect the DD design to protect against the effects of these errors.

Finally, there is the possibility of reverse causality, where previous conditions or legislation in Ontario brought about the decision for policy change and independently affected subsequent screening outcomes. We believe that due to federal oversight establishing guarantees for reasonable access to care, harmonized training of healthcare professionals, and conditional transfer payments from the federal government to provinces [17], the concerns of heterogeneity of health services delivery across provinces are dramatically reduced and the likelihood of endogenous factors driving policy change diminished. In Canada, the most severe disparities in health services delivery occur along north–south lines [17] which is in partly why the three Canadian territories were excluded from the analysis. Furthermore, that *ColonCancerCheck* utilizes previously established national screening guidelines common to all of Canada [7-9], and two other provinces in addition to Ontario are adapting similar programs, further lessens this concern.

## Conclusions

Our use of a quasi-experimental approach to derive a causal measure of the impact of the CRC screening program *ColonCancerCheck* from CCHS microdata files demonstrates the effectiveness of the program in increasing past-year screening by FOBT up to the end of 2009. The results from this study are largely generalizable to other Canadian provinces [46] in addition to other countries [47,48] which, in many cases, have commonalities in target populations and screening strategies. Insofar as *ColonCancerCheck* has been successful at increasing rates of screening with FOBT in the asymptomatic average risk population, this study provides evidence that the educational, instructional, and other outreach strategies adopted by the program have been successful. At this point, we are unable to make conclusions as to the effect of the program on use of endoscopy as it is unlikely for an appreciable effect to be observed so soon after program initiation. Another important caveat to this conclusion is that jurisdictions with varying pre-intervention screening levels may experience different degrees of outcomes, so the generalizability of the effect measure should be interpreted with caution. We cannot speculate on the long-term effects of *ColonCancerCheck* given that it is ongoing [23], that its ability to induce sustained patterns of preventive screening is unknown, and that it must accommodate updated screening recommendations [10]. Updated evidence of the protective effect of flexible sigmoidoscopy [49] and the improved sensitivity of [50] and test adherence to [51] fecal immunochemical tests is encouraging. The conclusions of the current study suggest that similar programs will be capable of promoting these procedures in the average risk population. Among the most important questions in the effectiveness of the program is its ability to reduce morbidity and mortality from CRC. This is not addressed by this study and presents an important avenue for future research examining mortality, costs, stages of cancer at detection and other important outcomes. Given previous evidence of the effectiveness of CRC screening program-like interventions in reducing CRC mortality [11] our results provide some level of optimism that this aim will become fulfilled.

## Abbreviations

CRC: Colorectal cancer; FOBT: Fecal occult blood test; PCP: Primary care provider; CCHS: Canadian community health survey; DD: Difference-in-differences design; DDD: Difference-in-difference-in-differences design; CCC: ColonCancerCheck; ON: Ontario; Post: Post Intervention Period.

## Competing interests

We declare no financial or non-financial conflicts of interest. Sponsors and/or supporters of the research had no contractual rights to read or approve the manuscript or review and comment on the manuscript.

## Authors’ contributions

TJC contributed substantially to conception and design, and the acquisition of data, analyzed and interpreted the data, drafted the article, and revised it critically for important intellectual content. ECS contributed substantially to conception and design, acquisition of data, interpretation of analysis results, and revised the article critically for important intellectual content. MJS contributed substantially to conception and design and revised the article critically for important intellectual content. All authors read and approved the final manuscript.

## Pre-publication history

The pre-publication history for this paper can be accessed here:

http://www.biomedcentral.com/1472-6963/13/449/prepub
